# Dragon boat racing and health-related quality of life of breast cancer survivors: a mixed methods evaluation

**DOI:** 10.1186/1472-6882-13-205

**Published:** 2013-08-05

**Authors:** Heather A Ray, Marja J Verhoef

**Affiliations:** 1 Department of Physical Education and Recreation, Mount Royal University, Calgary, AB, Canada; 2 Department of Community Health Sciences, University of Calgary, Calgary, AB, Canada

## Abstract

**Background:**

Breast cancer survivors who participate in physical activity (PA) are reported to experience improved health-related quality of life (HRQOL). However, the quantitative research exploring the relationship between the team-based activity of dragon boat racing and the HRQOL of breast cancer survivors is limited. Given the rising number of breast cancer survivors, and their growing attraction to dragon boating, further exploration of the influence of this activity on HRQOL is warranted.

**Methods:**

This study is designed to: 1) quantitatively assess whether and how breast cancer survivors’ participation in a season of dragon boat racing is related to HRQOL and 2) qualitatively explore the survivors’ lived experience of dragon boating and how and why this experience is perceived to influence HRQOL. A mixed methods sequential explanatory design was used with the purpose of complementing quantitative findings with qualitative data. Quantitative data measuring HRQOL were collected at baseline and post-season (N = 100); semi-structured qualitative interviews were used to elicit a personal account of the dragon boat experience (N = 15).

**Results:**

Statistically significant improvements were shown for HRQOL, physical, functional, emotional and spiritual well-being, breast cancer-specific concerns and cancer-related fatigue. A trend towards significance was shown for social/family well-being. Qualitative data elaborated on the quantitative findings, greatly enhancing the understanding of how and why dragon boat racing influences HRQOL.

**Conclusions:**

The use of a mixed methods design effectively captured the complex yet positive influence of dragon boating on survivor HRQOL. These findings contribute to a growing body of literature supporting the value of dragon boat racing as a viable PA intervention for enhancing survivor HRQOL.

## Background

Breast cancer is the most prevalent cancer amongst Canadian women. An estimated 23,800 women will be diagnosed in 2013, with 5,000 associated deaths occurring in Canada [1]. Advances in technology, more aggressive medical treatments and earlier detection rates have contributed to an estimated 5-year relative survival rate of 88% for those diagnosed with breast cancer [1-3]. Breast cancer and its treatment is associated with a host of negative side effects, long-term symptoms and troubling changes in appearance that can hinder health-related quality of life (HRQOL). For purposes of this research, HRQOL refers to the extent to which one's usual or expected physical, emotional, and social well-being are affected by a medical condition or its treatment [4]. Of the disease consequences experienced by breast cancer survivors, those most commonly reported to reduce HRQOL include fatigue, insomnia, pain, arm morbidity, lymphoedema, depression and fear of recurrence [5-10].

Given the rising number of survivors facing on-going challenges, the development and evaluation of interventions to prevent and/or reduce adverse outcomes, promote longevity and improve survivor HRQOL is of great importance [11-13]. A number of interventions are available to improve psychosocial outcomes such as cognitive-behavioural therapies, information and educational strategies, counselling, psychotherapy and support groups [14-16]. These interventions do not address the physical challenges to HRQOL that may also impede survivor psychological morbidity and social well-being [14,16]. To address survivor psychosocial, as well as physical and functional concerns, a growing body of literature supports physical activity (PA) as an effective intervention strategy [17-39].

A mode of PA that is growing in popularity amongst breast cancer survivors is the team-based activity of dragon boat racing. Dragon boating is said to have originated as a Chinese ritual during the 4th Century BC and has evolved into a major sporting event [40]. As described by the International Dragon Boat Federation this activity involves strenuous, repetitive upper body exercise, as 18 to 22 team-mates propel a 12 meter long boat through the water [41]. The beneficial nature of this activity has caught the attention of people worldwide as demonstrated by the influx of recreational and competitive teams existing globally.

Qualitative literature describes an increased physical, emotional, social, and to a lesser degree, spiritual well-being of survivors who paddle [42-49]. However, the quantitative literature specific to HRQOL of breast cancer survivors who dragon boat is limited. Employing the SF-12, a generic HRQOL measure, one quantitative study has been performed in this area reporting women who participate in a season of dragon boating experience enhanced mental and physical well-being [50].

Given the rigor of quantitative methods and the subjective information gained through qualitative inquiry, the use of mixed methods is increasingly common in the assessment of HRQOL [51]. The logic being that neither quantitative nor qualitative methods alone have the ability to capture the complexity of HRQOL; when combined these methodologies foster a greater understanding of the experience [52,53]. Being the first in this field to embrace the benefits of combining quantitative and qualitative methodologies, the purpose of this research is to quantitatively examine the relationship between participating in a season of boating and survivor HRQOL; and to qualitatively explore the survivors’ lived experience of dragon boating as to how and why paddling is perceived to influence HRQOL.

## Methods

This study is designed to address the following research questions: 1) Do breast cancer survivors who participate in a season of dragon boat racing experience a change in HRQOL from baseline to post-season, as assessed by valid and reliable quantitative HRQOL measures? If so, what degree of change is reflected within physical, functional, emotional, social/family and spiritual well-being?; 2) What does dragon boat racing mean to breast cancer survivors and what are their perceptions of how this experience has influenced their HRQOL? Specifically, how do they describe (using qualitative inquiry) this experience as well as any changes related to the physical, functional, emotional, social/family and spiritual well-being?

This study is a mixed methods sequential explanatory design consisting of two distinct phases. Phase I was given research priority using paired measurements to quantitatively examine whether breast cancer survivors who participate in a season of dragon boating experience a change in HRQOL from baseline to post-season. Phase II consisted of qualitative interviews allowing us to determine themes underlying why survivors perceived paddling to influence HRQOL, which was difficult to capture quantitatively. The quantitative and qualitative findings were combined during the interpretation of the data, where qualitative themes were used to better explain and elaborate on the quantitative findings.

The conjoint Health Research Ethics Board at the University of Calgary granted ethics approval for this study (Reference number 23000). Personal identifiers have been removed or disguised to preserve anonymity.

### Quantitative methods-phase I

#### Participants

A consecutive sample of breast cancer survivors meeting the following eligibility criteria: 1) at least six months post-treatment (common pre-requisite for team participation), 2) an active team member for the 2010 paddling season within British Columbia, Canada and 3) being able to read and speak English. One hundred and sixteen breast cancer survivors, from 14 dragon boat teams located in British Columbia, volunteered to participate in the baseline data collection.

#### Data collection

Email addresses as available to the public on the Abreast in a Boat Society website were used to contact team managers for the purpose of recruitment [54]. Managers agreeing to take part in the research distributed study documents to team members who consented to participate in Phase I by providing an email or mailing address to the researchers. Study participants were given the option to receive the quantitative surveys via Survey Monkey or to complete print-based questionnaires delivered by mail. Four of the women completed the mail versus on-line survey. On-line survey administration was based on considerations of economy, convenience, and accessibility to respondents.

#### Data collection instruments

The baseline quantitative survey included demographic data, disease characteristics and the Functional Assessment of Cancer Therapy-Breast (FACT-B) [55], Functional Assessment of Chronic Illness Therapy-Spiritual Well-Being Scale (FACIT-Sp12) [56], and the Functional Assessment of Cancer Therapy-Fatigue Scale (FACIT-Fatigue) [57-59]. The FACT-B is a 37-item compilation of questions divided into five subscales including physical, functional, emotional, social/family and breast cancer-specific concerns. The FACIT-Sp12 is a 12-item subscale assessing two sub-domains of spiritual well-being including peace/meaning, and faith. The FACIT–Fatigue Scale is 13-item subscale measuring an individual’s level of fatigue during usual daily activities over the past week. All study instruments have been tested for validity and reliability [55]–[59]. Minimally important differences (MID’s) have been determined for the FACT-B and FACT-Fatigue [60]. MID’s are clinically significant changes that have been determined to be the smallest score differences from baseline measurement to subsequent measurements that are perceived as important to the patient and practitioner [60,61]. In addition to the FACT-B, FACIT-Sp 12 and FACIT-Fatigue, the post-season survey included: disease characteristics and participation-related data such as number of seasons, length of season, frequency of on-the-water training, number of competitions and number of social events attended over the year.

#### Data analysis

The quantitative data were analyzed using SAS version 9.2 [62]. In the case of missing data, scores were prorated when more than 50% of the items on each scale had been completed. If a participant completed less than 50% of the items, the participant data were not included in the quantitative analysis [63]. Descriptive statistics and frequencies were used to describe the characteristics of the study sample. Paired sample t-tests were used to compare the mean change scores in HRQOL from baseline to post-season on FACT-B, Physical, Functional, Emotional, and Social/Family Well-Being subscales, FACIT-Sp12 and FACIT-Fatigue. Analysis of variance (ANOVA) was used to compare the difference between HRQOL scores at baseline and number of seasons of dragon boat participation.

### Qualitative methods-phase II

#### Interview participants

Interviewees were recruited from Phase I participants. Team members interested in participating in an interview were asked to email the researchers directly as requested on the post-season survey in Phase I.

#### Data collection

Due to the geographic distance between the researcher and the study participants, semi-structured interviews were carried out via telephone. Each audio recorded interview lasted between 30 and 60 minutes. Open-ended questions were used to elicit a personal account of the dragon boat experience including how they became involved with paddling, if and how they felt the experience may have influenced their HRQOL and challenges they may have experienced (See the Qualitative interview guide section). The researcher encouraged an open dialogue and supported the participants in sharing other aspects of their dragon boat racing experience as they deemed important.

#### Qualitative interview guide

1. How did you first get involved in dragon boat racing?

2. How long have you been involved with an organized dragon boat team?

3. Please tell me about any physical changes you have experienced as a result of paddling over this season? How do you feel these changes may have influenced your quality of life?

4. Please describe how you feel participation in this paddling season may have affected your emotional well-being? How do you believe this may have influenced your quality of life?

5. In what way do you feel your involvement with dragon boat racing and your team may have affected you socially? How do you believe this may have influenced your quality of life?

6. How has the participation in dragon boating this season influenced how you view your life from a spiritual perspective? How do you believe this may have influenced your quality of life?

7. How would you describe the experience of dragon boat racing this season in regards to how it may have affected your overall quality of life?

8. During this conversation, you have discussed several advantages of participating in dragon boat racing. What would you describe as being the greatest benefit(s) you have experienced in regards to positive changes in your quality of life related to paddling?

9. In the unfortunate circumstance of a recurrence with a team member, how do you feel this affects the team members? Do you feel this experience may act as a reminder of cancer and/or deterrent from being on a dragon boat team with breast cancer survivors?

10. What challenges have you experienced being involved with dragon boat racing and being a part of this team activity?

11. Are there any other experiences or comments you would like to share that you feel have not been addressed in this interview, relating to your dragon boat experience and its influence on your quality of life?

#### Data analysis

Audio recordings were transcribed verbatim. Content analysis of the qualitative data was performed following the linear, hierarchical, 6-step approach proposed by Creswell: 1) organizing the data for analysis, 2) reading all data to gain meaning, 3) coding the data, 4) describing themes, 5) determining how themes will be represented, and 6) interpretation of the data [64]. A list of codes related to each research question was compiled as transcripts were reviewed. Themes were then identified and described within and across participant experiences and perceptions. The final step in the qualitative content analysis was the assessment and interpretation of the themes, thus, giving meaning to the lived experience of dragon boat racing as portrayed through the narrative data [65].

To enhance credibility of the findings, member checking was performed where themes were brought back to participants for verification of interpretation [64,66]. Eight participants provided feedback and verified the accuracy of the findings. The remaining interviewees were considered unreachable after a second follow-up email resulted in no response. Dependability was established through the systematic analysis used in the qualitative phase of the study. Data collection and data analysis procedures are described to enable other researchers to judge the transferability of findings and/or to facilitate study replication. Confirmability was addressed through inter-coder agreement where both researchers reviewed the content analysis procedures and independently reviewed and coded the interview transcripts [64,66].

## Results

### Quantitative results-phase I

#### Response rate

One hundred sixteen breast cancer survivors completed the baseline quantitative survey. One hundred women were included in the post-season analysis giving an 86.2% study retention rate. The 16 women withdrawing after the baseline assessment did not respond to the four contacts made by the researchers regarding participation in the post-season survey.

#### Demographic, disease and team-related characteristics

More than half of the sample (54.9%) was at least 60 years or older and 75.5% were married or in a stable relationship. More than 5 years had passed since diagnosis for 52.0% of participants. Of participating women, 34.3% reported a diagnosis of non-invasive carcinoma, invasive ductal carcinoma (46.1%), invasive lobular carcinoma (39.8%) or other diagnoses (9.8%). At baseline 84.4% of women had Stage 0 or Stage I cancer. All women had surgery, radiation (69.6%), chemotherapy (57.8%), aromatase inhibitors (22.6%), tamoxifen (47.0%) and/or other treatments (9.8%). The most commonly reported comorbidities in the sample were arthritis (52.9%) and osteoporosis (25.5%). Six women (5.9%) reported a recurrence, metastasis or other change during the season. This was the first season of racing for 21.2% of women and 56.6% had participated in four or more seasons. Most women (90.0%) participated in two or more on-water training sessions per week and 74.3% participated in 2 or more competitions over the dragon boat season. Three-quarters of the sample (75%) participated in three or more team-related social events over the year (Table 1).

**Table 1:** Demographic, disease and team-related characteristics (N = 100)

**Baseline**	**%**	**Post-season**	**%**
**Age (years)**		**Comorbidities**	
Under 40	2.0	Osteoporosis	25.5
40–49	9.8	Arthritis	52.9
50–59	33.3	Heart disease	15.7
60–69	44.1	Diabetes	3.9
Over 70	10.8		
**Relationship Status**		**Change in Cancer Status over Season**	
Single	8.8	Recurrence	3.9
Married/stable Relationship	75.5	Metastasis	1.0
Separated	2.9	Other	1.0
Divorced	11.8		
Other	1.0		
**Employment Status**		**Number of Seasons Paddling**	
Employed Full-time	24.5	1 season	21.2
Self-Employed	7.8	2 seasons	10.1
Part-time/Seasonal	15.7	3 seasons	12.1
Unemployed	1.0	4 or more	56.6
Homemaker	2.9		
Retired	43.1		
Other	3.9		
**Time Since Diagnosis**		**On-Water Training Sessions Per Week**	
Less than 3 years	23.5	1 session	10.1
3–4 years	9.8	2 sessions	79.2
4–5 years	14.7	3 sessions	10.0
More than 5 years	52.0	4 or more	1.0
**Cancer Type**		**Weeks of Paddling in 2010 Season**	
Non-Invasive Carcinoma	34.3	Less than 8 weeks	10.1
Invasive Ductal Carcinoma	46.1	9–12 weeks	6.1
Invasive Lobular Carcinoma	9.8	13–14 weeks	14.1
Other	9.8	15–16 weeks	10.0
		More than 17 weeks	59.6
**Cancer Stage**		**In-Season Competitions**	
0	68.8	None	6.9
I	15.6	1 competition	18.8
II	9.4	2 competitions	23.8
III	4.2	3 competitions	10.9
IV	2.1	4 or more	39.6
**Past Treatment**		**Team-Related Social Events**	
Surgery	100.0	1 per year	7.0
Radiation	69.6	2 per year	18.2
Chemotherapy	57.8	3 per year	19.5
Aromatase Inhibitors	22.6	4 or more per year	56.1
Tamoxifen	47.0		
Other	9.8		

#### Change in HRQOL

The mean change scores from baseline to post-season were statistically significantly (p < 0.05) different from zero for: FACT-G (p = <0.001), FACT-B (p = <0.001), Physical Well-Being (p = 0.003), Functional Well-Being (p = 0.019), Emotional Well-Being (p = <0.001), Spiritual Well-Being (p = 0.036), Breast Cancer-Specific Concerns (p = 0.006) and FACIT-Fatigue (p = <0.001). Mean score changes which were not statistically significantly different from zero (p < 0.05) include: Social/Family Well-Being (p = 0.059) and the FACIT-Sp subscale assessing meaning and peace (p = 0.204) (Table 2).

**Table 2:** Mean HRQOL change scores, paired T-tests

**Measure**	**N**	**Mean change (SD)**	**95% confidence interval**	**ES**	**p-value**
FACT-G	100	2.77 (7.09)	1.37, 4.18	0.22	<0.001
FACT-B	100	3.73 (8.90)	1.96, 5.50	0.23	<0.001
Physical Well-Being	100	0.51 (1.69)	0.18, 0.85	0.16	0.003
Functional Well-Being	100	0.77 (3.23)	0.13, 1.41	0.18	0.019
Emotional Well-Being	100	0.84 (2.35)	0.38, 1.31	0.25	<0.001
Social/Family Well-Being	100	0.64 (3.37)	−0.02, 1.31	0.13	0.059
Breast Cancer Concerns	100	0.96 (3.41)	0.28, 1.63	0.18	0.006
FACIT-Sp12	100	1.03 (4.82)	0.07, 1.98	0.14	0.036
FACIT-Sp Meaning/Peace	100	0.47 (3.71)	−0.26, 1.21	0.09	0.204
FACIT-Sp Faith	100	0.55 (2.64)	0.03, 1.08	0.13	0.038
FACIT-Fatigue	100	1.64 (4.72)	0.70, 2.57	0.19	<0.001

Effect Size (ES) = Mean Change/SD at Baseline.

p-value is Testing Null Hypothesis that Change is Zero.

#### Baseline HRQOL and number of seasons

ANOVA testing showed women who had participated in more than one season of dragon boat racing had better baseline FACT-B (p = 0.016), physical well-being (p = 0.013), functional well-being (p = 0.009), and FACIT-Fatigue (p = 0.007) scores, than women in their first season.

### Qualitative findings-phase II

#### Demographic, disease and team-related characteristics

Fifteen consenting women, representing 9 different teams participated in qualitative interviews. Fourteen of the women were age 50 or older. Most women (13) were currently married or in a stable relationship. More than 5 years had passed since diagnosis for eight of the women interviewed. Of participating women, five reported a diagnosis of non-invasive carcinoma, invasive ductal carcinoma (5) or invasive lobular carcinoma (5). At baseline eleven women had Stage 0 or Stage I cancer. All women had surgery, radiation (11), chemotherapy (9), aromatase inhibitors (4) and/or tamoxifen (8). This was the first season of racing for three of women and seven had participated in four or more seasons. Fourteen women participated in two or more on-water training sessions per week and ten participated in 2 or more competitions in the dragon boat season. Nine of interviewees participated in three or more team-related social events over the year.

### Qualitative themes

Reflective of the main concepts being explored, seven themes resulted from the qualitative data analysis. Quotes are only included if they add substantially to the understanding of the themes.

#### Theme 1: physical fitness and lifestyle

All women shared the positive experience of having improved cardiovascular endurance, more strength in arms, shoulders, legs and core, and the positive impact of improved fitness levels on daily functioning. Team members described how the physical benefits of paddling such as improved strength, stamina and energy levels helped them function more effortlessly throughout the day. Participation in dragon boat racing was also perceived to inspire women to be conscious of living a well-rounded and healthy lifestyle:

It has made me much more aware of my health because I am engaged in a physical activity and it has made me much more aware of things like my diet, exercise levels and stress management. Team members are always sending you tips that they learn about improving your health that I find very interesting and helpful. (P01)

#### Theme 2: emotional strength and reduced stress

Dragon boat racing was a catalyst for improved emotional strength and enhanced their ability to “slay the dragon”. Women felt better about themselves, both physically and mentally, where higher self-esteem was attributed to greater self-acceptance and self-confidence. Self-acceptance was supported by choosing to live a healthier lifestyle and having an improved body-image. Greater self-confidence was portrayed as being two-fold; fostered by improved physical functioning, increased endurance, strength and better overall health; and by perceptions of mastery as associated with regaining control over ones body in combination with learning/improving paddling skills and technique:

I would definitely say that because of the women that I paddle with I am definitely not as hard on myself as I used to be and I like myself more. I feel that the strengthening, the conditioning and improving your technique help you feel emotionally strong, not feeling like ‘I don’t know if I can handle that or do that’. I now feel like I can do well almost anything. I do need to consider that I am not that young but knowing I can paddle has given me the confidence to go out and try new things. (P09)

Increased motivation to participate both during the dragon boat season and throughout the year was promoted by a combination of physical and psychological variables. Physically, participants spoke of appreciation for the many fitness and health benefits of active participation during the season, and also of the importance of maintaining fitness year-round. Psychologically, honouring the commitments made to oneself and to the team also acted as strong motivators for adherence. While there was agreement amongst the participants that facing a recurrence with a team-mate was emotionally difficult, a number of the participants shared how facing such a challenge provided them strength, promoted togetherness and offered support in facing their own fear of recurrence:

It reminds us all that we could have a recurrence, but I think it reminds us too, certainly for me, that I would have an immediate support network that I could count on. I am pretty proud of my crewmates and the love that they show and the capacity that they have for support… it is really encouraging to see how the rest of the crew reacts when somebody has got some test results they are worried about or they have something scary happening, and to see how the crew rallies around. (P02)

Dragon boating was described by many as acting as a buffer against stress that helped to offset worries and anxiety. Having a strong support network and becoming engrossed in the motion of paddling helped women stay in the ‘here and now’ instead of ruminating about the past or focusing on stressful events. Several women described their experience as being similar to doing meditation where the repetitive motion of paddling, finding unison of stroke with team-mates, and enjoying ‘being in the present’ was described as a powerful means of managing stress. Participants’ expressions of this experience included:

For stress levels I think it is great because you get out on the water and you forget. I don’t think of my job, I don’t think of cancer, I don’t think of any problems. Believe me, you are out there and you concentrate, you have got a lot of things you have to think about. You know, where your arms are, and your hands are, and your legs are, and your feet are, and how you are sitting and leaning out and where you are looking and you have got to concentrate. (P11)

#### Theme 3: social support network

Literally ‘all in the same boat’, the presence of physical and emotional comfort was emphasized as creating a safe environment in which to share personal experiences and thus facilitated feelings of acceptance, support and creating trust through familiarity. Participants spoke of experiencing a common bond with team mates who are in many ways much different from themselves, but who share a familiar experience of cancer and the common interest of dragon boating. Women found it comforting to have team mates who had experienced similar situations to which they could turn to for information and support:

I find there is a familiarity…with these women that I can’t quite define and it is not tangible but I feel something, more closer to them; I feel close to them for whatever reason and for reasons other than just we boat together and we do things together. There is something that as we talk or I get to know them as people I think, ‘I like that. I like that person. That person makes me feel good and I like being around them. I feel safer and I trust that person’. (P04)

While striving to represent good health, women talked about playing a role in supporting and motivating other breast cancer survivors to live life to the fullest. Also shared was the importance of creating public awareness of life after breast cancer, while shattering the negative stigma that surrounds the disease. Emphasis was placed on being living proof that these women not only survived breast cancer, but that they have gone on to live healthy, active and fulfilling lives:

I am a survivor of breast cancer and I always think how much that helps people because they are looking at someone who looks good and is fit…I think it is a wonderful, wonderful program because it takes away that nastiness that the word ‘cancer’ tends to have. Particularly if they don’t know anyone who has had cancer and they see this person in the bank or in the library and they think, ‘Oh she looks terrific’. (P15)

#### Theme 4: obstacles and deterrents

The described obstacles and deterrents were shared as sometimes having a negative influence on the social aspect of dragon boating, however most women believed the benefits gained from the experience outweighed such challenges. Some team members preferred dragon boat racing to be health and recreation focused with less competitive interest, while others liked it to be more competitively driven. As a result conflicts sometimes did arise, with most issues being resolved by making a team decision as to the level of desired competitiveness.

I really liked the paddling and I found I was a very poor paddler but that didn’t really bother me because I always want to try and learn new things. The coaching was fabulous, the women were very approachable, but there seemed to be a very strong emphasis on competition. If there had been a team closer to my home that was recreational based I would have been in that boat as much as I could have been. I think it probably would be a really positive experience if I were in that kind of mindset. I may not have been the right time or the right place for me… (P13)

The physicality of paddling was not viewed as an obstacle that would deter participation. Rather, the emphasis was placed on the challenge of committing the time to attend practices; and in some cases the demands of volunteering and fundraising were also identified as obstacles and/or deterrents. Team spirit, harmony and morale were also sometimes negatively impacted by personality conflicts amongst the team members. Although challenging, generally these issues were viewed as somewhat expected given the large number of team mates having differing personalities and backgrounds.

#### Theme 5: spiritual health and the new normal

The aspect of hope for a life after breast cancer was commonly shared by the women indicating a renewed appreciation for life, a restoration of hope for living a healthier life, being physically active, having fun, and being surrounded by a group of supportive team mates:

I think there is hopefulness, you know, in the future, but it is much more of a ‘try to live each day’ and try to make sure that most days are good. Whether it is religious or whether it be of a personal spiritual feeling, life can be good, and even when it is a hard it can be good. You think to yourself there is something good happening here on this team and how lucky I am to be with these wonderful people who have got such stamina and such strength and who are so happy and supportive. (P09)

The interaction with nature was expressed as being an external stimulus that was important for restoring a sense of peace and aliveness. All women described the joy of being in nature, the beauty, the smells, the sounds and the feeling of the paddles moving in unison going through the water. Described by one participant as creating a “Zen kind of feeling”; and another of how the anticipation of being outside gliding on the water took priority over the worries she may be having that day.

I think there is a real sense of peace that you get after you have done it because when you are in the boat you have to focus on what you are doing, and you focus on you and not what everybody else is doing. I find being on the water is that added benefit and it is very peaceful to be out there and I so look forward to it. It is addicting! …I just find it is a place for me to let go of everything and when on the water I am removed in some way from the land. I just find it is a place where I can get all together to myself. (P04)

Also shared was the challenge of trying to separate the spiritual growth that was attributed to the dragon boat experience, from that experienced by having had and survived cancer. Several women expressed how their spiritual well-being had been enhanced from a combination of dragon boating and surviving cancer, where neither experience could be thought of as a separate entity. Awareness of such ambiguity is described by the following breast cancer survivor:

…I feel very spiritual. In all fairness I don’t think it has all come from dragon boating as some of it has come from going through cancer and actually being lucky enough to survive. Allowing that experience to open your mind and to see yourself and your surroundings, helps you make the changes you need to do to feel better. I think that being with a group of women who share a common goal of paddling together as well wanting to thrive have both added to my spirituality… (P04)

#### Theme 6: enhanced HRQOL

The combined influence of dragon boat racing on the physical, emotional, social and spiritual well-being was described as enhancing total HRQOL:

I think being on the water is calming, it is beautiful, and it is just really wonderful. Being together is uplifting, it is a sunny, beautiful day, it is warm, it is glorious, you have worked, you are tired, you get out from the water, you are together, you are laughing, you are joking, you are putting out some physical effort and it lifts you up and it makes you feel really good…Even if we didn’t do our best and some of our times weren’t that great, it about the being together, in that moment out on the water. It is that you are out there, you are out in nature, you are out in the world and it really does make a big difference… it helps you appreciate and take hold of everything every day and you don’t take it for granted anymore. (P11)

#### Theme 7: uniqueness of dragon boat racing

Several facets of dragon boating were talked about by participants signifying the connotation of ‘all in the same boat’. A common perception amongst women expressed the uniqueness of dragon boat racing as having all team members coming together, working in unison with the same timing and rhythm in order to propel the dragon boat forward. Many women also shared that regardless of physical fitness, strength, paddling experience, or previous involvement with sport, everyone could participate:

We have people at all stages, like me who is fourteen years on and we had other people who had just been diagnosed when they came out to the first meeting. There is a whole range of experience there for everyone to draw on… This may not be for everybody, but everybody has a place on our team and it doesn’t matter about your age, your abilities or whether you want to go to festivals or not. If you are a breast cancer survivor then you have a place in our boat. (P01)

**Figure 1:**
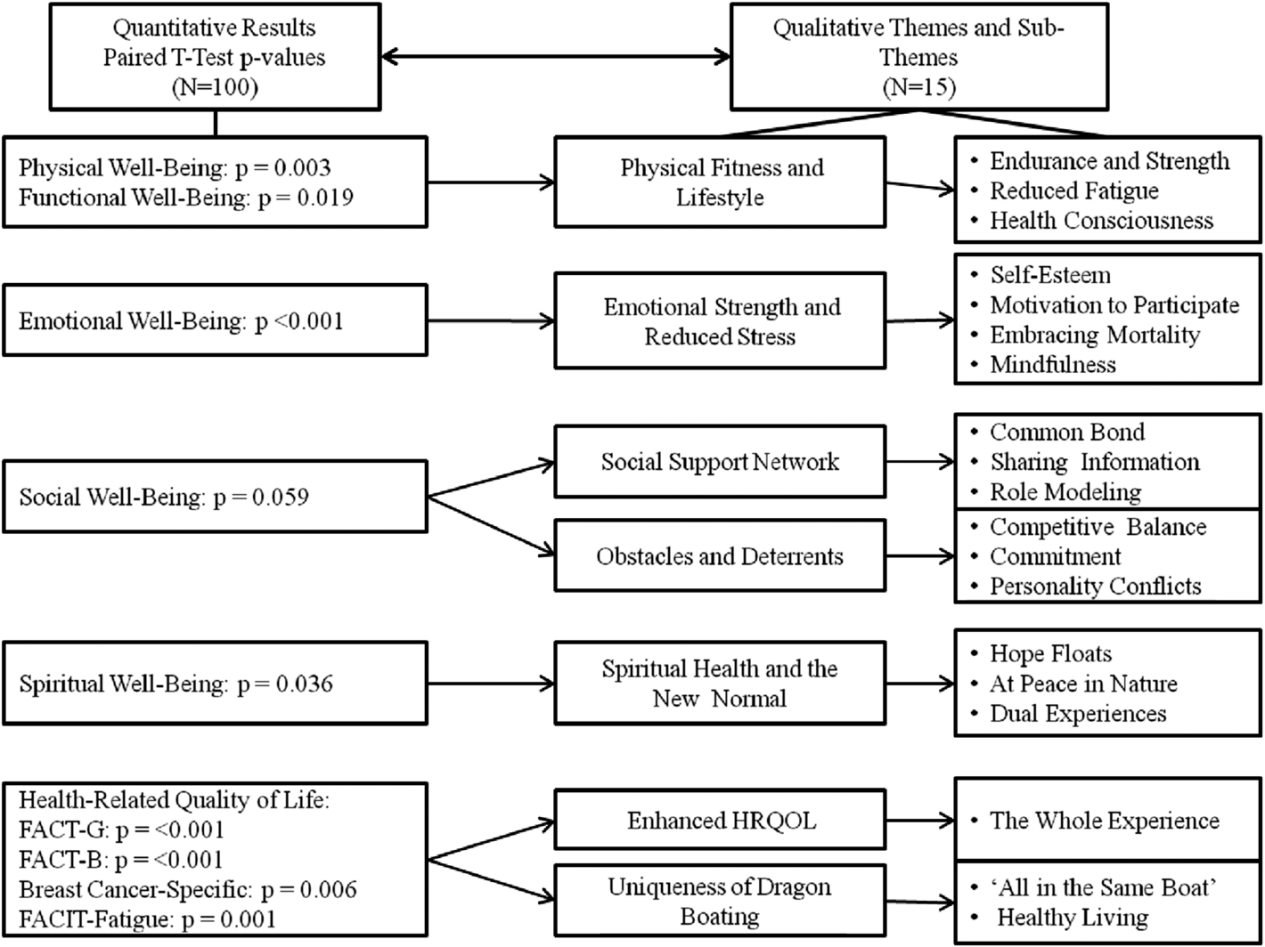
Synopsis of mixed methods findings.

As depicted in Figure 1, qualitative data provided richness and depth to the interpretation of both significant and non-significant quantitative findings and were fundamental in explaining why women perceived dragon boat racing to influence HRQOL.

## Discussion

### HRQOL

Findings support the benefit of dragon boating as a PA on breast cancer survivor HRQOL, physical, functional, emotional and spiritual well-being, breast cancer-specific concerns and cancer-related fatigue [17-39]. The change in HRQOL did not reach the established MID’s representing a clinically significant change. This finding is not surprising given this was a physically active sample, many of whom being long-term survivors with already high baseline HRQOL. Women who had participated in one or more seasons of boating also show better baseline HRQOL, physical and functional well-being, and lower cancer-related fatigue than women in their first season. Participants reporting chemotherapy (57.84%), radiation (69.6%), Aromatase Inhibitors (22.6%) and/or Tamoxifen therapy (47.0%) may have experienced common side effects such as weight gain, deconditioning and cancer-related fatigue [67]. Given that such concerns may be amenable to a PA intervention, it is possible that women undergoing treatment may have experienced a greater improvement in HRQOL in comparison to those not experiencing acute or long-term treatment effects. As a whole, qualitative findings echo those in the published dragon boating literature, supporting the benefits of paddling to survivor HRQOL and well-being [42-49].

### Physical and functional well-being

Participation in a season of dragon boating may be beneficial in reducing the intrusive symptoms of breast cancer and treatment side effects impacting survivor physical and functional well-being. Survivors expressed contributing factors to include greater cardiovascular fitness, improved muscular strength and endurance, greater stamina, reduced levels of cancer-related fatigue, and striving to live a healthier lifestyle year-round. This is a notable finding recognizing that physical and functional health may also influence emotional and social well-being outcomes [68].

### Emotional well-being

Comparable research to formulate conclusive statements regarding the significant improvement in emotional well-being is not available; however our findings support the broad PA and survivorship literature being indicative of women experiencing reduced feelings of sadness, hopelessness, anxiety, worry and fears surrounding their illness [69]. Emotional strength was attributed to higher self-esteem, self-acceptance, self-confidence and increased motivation to be physically active year-round. Being mindful of the moment was pivotal in stress management, as encouraged by the rhythm of paddling, being in unison with team mates, and striving to focus on each paddle stroke. Importantly, the mutual support system offered through team dynamics was fundamental in helping women manage stress by feeling accepted, needed and worthy.

### Social/family well-being

A non-significant improvement for social/family well-being was a surprising outcome given that women felt allegiance to a strong support network and believed such support improved HRQOL and social well-being. Acknowledging the inter-relatedness of the HRQOL domains is one explanation for not reaching statistical significance in social well-being. Social support was a catalyst for improving emotional well-being via improved emotional strength and as a result it is plausible that the positive influence of social support may have been reflected more strongly in the significant improvement shown for emotional well-being. Despite the many positive aspects of the dragon boat experience, personality conflicts, differences in competitive drives and varying commitment levels were portrayed as creating social discord. These challenges may have diminished the social experience consequently influencing social well-being outcomes.

### Spiritual well-being

Qualitative findings support the significant difference in the FACIT-Sp12 and for the Faith subscale, denoting a positive change in spiritual well-being in response to participation in the dragon boat racing season. In agreement with qualitative dragon boat literature, women shared how spiritual well-being was enhanced by gaining greater hope for the future, a renewed appreciation for life, and enjoyment from being in nature [43,44,46-48]. Based on the interpretation of the qualitative data, several possible explanations are proposed to explain the non-significant change on the FACIT-Sp12 subscale assessed meaning/peace. Women more comfortably described the physical changes, emotional strength and presence of social support as being tangible effects, which were easier to gauge in association with their participation dragon boating. Consequently, the elusiveness of spiritual growth and the challenge of interpreting the meaning of dragon boat racing, in isolation from the broader cancer experience, may have influenced participants’ responses on the FACIT-Sp12 meaning/peace subscale.

### Breast cancer-specific concerns

A range of breast cancer-specific concerns are shown to impede return to pre-illness functioning such as being more self-conscious of how one is dressed, swollen or tender arms, pain, shortness of breath, feeling sexually unattractive, feeling less like a woman, and concerns related to changes in body weight, hair loss and the effects of stress on ones illness [55]. Many felt dragon boating helped to improve and/or alleviate many of their concerns, where the feeling of ‘all in the same boat’ encouraged participants to be more accepting of their body and helped to further empower them.

### Cancer-related fatigue

Given that cancer-related fatigue affects approximately 70-90% of all cancer survivors the significant improvement demonstrated on the FACIT-Fatigue is a prominent finding [70]. Reduced fatigue levels were experienced in response to paddling and achieving greater fitness, endurance and stamina. Although no comparable data exist within the dragon boat racing literature, this finding does support the positive role of PA during or following treatment for reducing the severity of cancer-related fatigue [17,26,35-39,71].

### Strengths and limitations

In addition to an 86.2% retention rate, this study provides the first quantitative assessment of the multi-dimensional relationship between dragon boat racing and HRQOL, well-being and cancer-related fatigue of breast cancer survivors. Using a mixed methods design this study is also the first to use qualitative data to enhance the interpretation of the quantitative findings, to provide a broader understanding of the survivors’ dragon boat experience. We recognize there are several limitations in this research. Not having a control group preventing the evaluation of causality. Given that the majority of participants (84.4%) reported Stage 0 and Stage I cancer, combined with the fact that this cohort is characteristically viewed as being highly active and motivated women, their experiences cannot be generalized to the broader population of breast cancer survivors. The original intent of the study design was to analyze the quantitative data before the qualitative interview data were collected. This was not possible as season-end dates varied between teams, making it impossible to analyze the quantitative data prior to implementing the interview schedule.

### Recommendations

Findings from this study suggest that higher baseline HRQOL may be associated with more years of paddling experience. Therefore, future research would benefit from employing a follow-up study with novice members to explore the relationship between dragon boat racing, and long-term HRQOL outcomes. Participation in dragon boating may not be ideal for all women, as each woman has different circumstances, a different medical history, and different goals and interests. Therefore, employing comparative effectiveness research (CER) is also recommended for future research.

## Conclusions

This mixed methods study has shed light on the dragon boat experience, suggesting that participation in a season of boating is associated with improved HRQOL, physical, functional, emotional and spiritual well-being, breast cancer-specific concerns and cancer-related fatigue. The interpretation of significant and non-significant quantitative findings was greatly enhanced with the broadened perspective of qualitative themes. These findings contribute to a growing literature supporting the benefits of dragon boat racing, bringing breast cancer survivors another step closer to having a PA option that may help them address challenges to HRQOL, while encouraging them to thrive during survivorship.

## Competing interests

The authors declared that they have no competing interest.

## Authors’ contributions

HAR conceived and conducted this study. MJV provided feedback in each phase of the study and helped draft the manuscript. Both authors read and approved the final manuscript.

## Pre-publication history

The pre-publication history for this paper can be accessed here:

http://www.biomedcentral.com/1472-6882/13/205/prepub
